# Meteorological Register

**Published:** 1834

**Authors:** Joseph Ray


### VII. Meteorological Register.

With the subjoined abstract, we commence the publication of a register of the state of the Thermometer, Barometer, Winds, Clouds, and Rain, kept at the Woodward High School, in the northern part of this city, lat. 39°.6′ and long. W. from Greenwich 84°.22′. The observations are made by Joseph Ray, M. D. one of the professors of that flourishing Institution; who to his professional attainments, adds a knowledge of Mathematics, Astronomy, and Physical Geography, and is, therefore, likely to make observations on which reliance may be placed.

### Abstract of Observations.

| Thermometer. | Jan. | Feb. | March. |
|---|---|---|---|
| Mean temperature | 28°.78 | 45°.29 | 43°.76 |
| Maximum | 62°. | 66°. | 70°. |
| Minimum | 0. | 22°. | 20°. |
| Range | 62°. | 44°. | 50°. |
| Barometer. | | | |
| Mean height | 29.473 | 29.278 | 29 339 |
| Maximum | 29.800 | 29.600 | 29.770 |
| Minimum | 28.260 | 28.630 | 28.920 |

Remarks.—The month of January was 11 degrees colder than the same month of the preceding year. The month of February was remarkable for regular temperature and pleasant weather; the number of clear days being 16 and that of the cloudy 12. In March the number of clear days was only 11. January, this month the direction of the wind was West 21 days, South 3 days and the remaining part of the month North and North-East. The whole depth of rain in this month was 3.87 inches. During the first two months of the year, no regular account was kept of the direction of the wind or the quantity of rain, this will be done in future. JOSEPH RAY, M. D.

